# Glycogen Storage Disease Type I With Hypercalcemia in an Infant: A Case Report

**DOI:** 10.7759/cureus.46987

**Published:** 2023-10-13

**Authors:** Aziza Elouali, Chaimae N'joumi, Amal Bennani, Maria Rkain, Abdeladim Babakhouya

**Affiliations:** 1 Department of Pediatrics, Faculty of Medicine and Pharmacy of Oujda, Mohammed First University, Oujda, MAR; 2 Department of Anatomic Pathology, Faculty of Medicine and Pharmacy of Oujda, Mohammed First University, Oujda, MAR; 3 Department of Pediatrics, Mohammed VI University Hospital, Oujda, MAR; 4 Pediatric Gastroenterology, Mohammed VI University Hospital Center, Oujda, MAR

**Keywords:** glycogen storage disease type i, case report, metabolic decompensation, hypertriglyceridemia, hypercalcemia, glycogen storage disease

## Abstract

Glycogen storage disease type I (GSDI) is an uncommon condition resulting from a deficiency or absence of glucose-6-phosphatase, a key enzyme in regulating blood glucose levels. In this report, we describe a two-month-old girl diagnosed with GSDI who presented to the emergency department in a tertiary care hospital for irritability, excessive crying, and hyperventilation. She was found to have hepatomegaly and hypoglycemia. Laboratory investigations showed high levels of triglycerides, lactic acid, uric acid, and calcium. The combination of hypertriglyceridemia, hypoglycemia, and hepatomegaly should alert neonatologists and pediatricians to consider GSDI in the diagnosis. Hypercalcemia arose as an unknown problem in GSDI patients and should be considered during acute attacks.

## Introduction

Glycogen storage disease type I (GSDI) is an autosomal recessive disorder of glycogen metabolism affecting one in 100,000 live births [1]. It is the most common subtype of hepatic glycogen storage diseases (GSDs). Two main subtypes are distinguished. In classic type Ia GSD (von Gierke disease), glucose-6-phosphatase is deficient. Type Ib is due to an impaired glucose-6-phosphate transporter (G6PT) activity [2]. Patients with GSDI have an enlarged liver, a recognizable "baby face," growth retardation, and chronic fatigue. Laboratory findings suggestive of GSDI include hypoglycemia, lactic acidosis, hypertriglyceridemia, and hyperuricemia [3]. Here, we present a case report of a two-month-old infant girl with hepatomegaly, severe lactic acidosis, hypertriglyceridemia, hyperuricemia, hypoglycemia, hypercalcemia, and bilateral nephrocalcinosis, who was diagnosed with GSDI by liver biopsy.

## Case presentation

**Figure 1:**
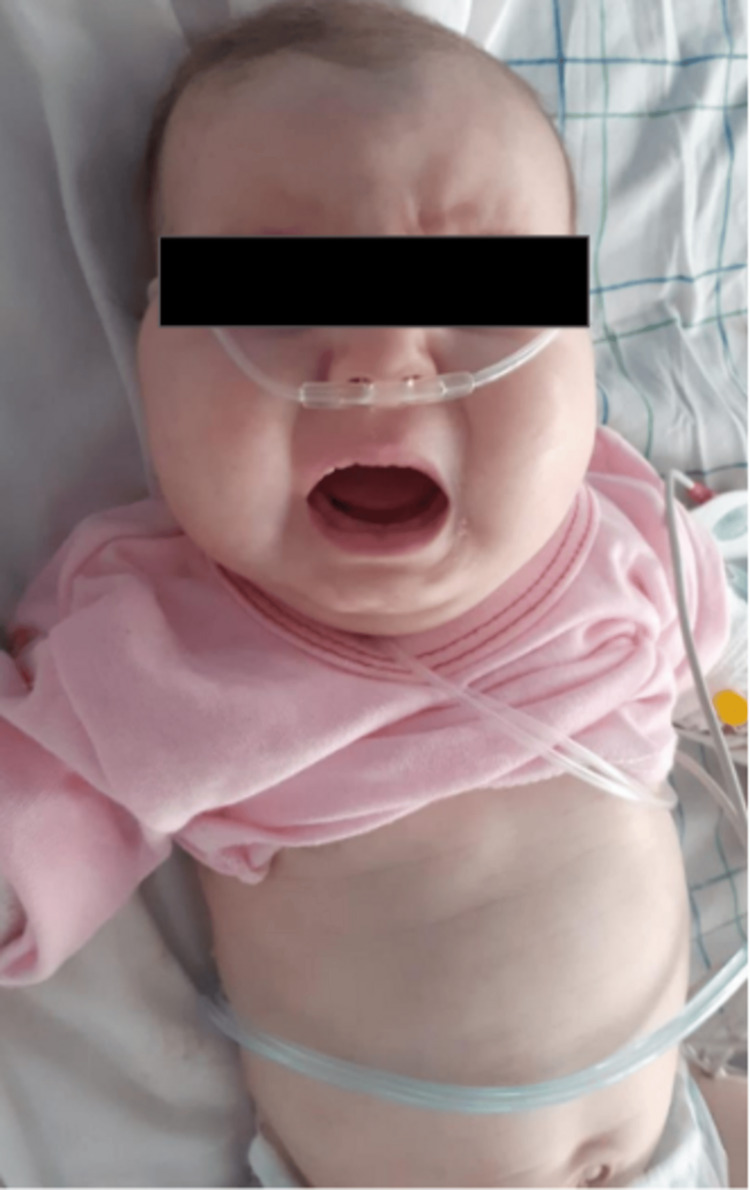
Image showing our baby with features of von Gierke’s disease: a characteristic doll-like, fatty cheeks and protuberant abdomen

A two-and-a-half-month-old female patient was referred by a private pediatrician to our emergency department of pediatrics with a three-day history of irritability, excessive crying, and labored respirations. The baby was born at full term by normal spontaneous vaginal delivery with a birth weight of 3500 g, a head circumference of 35 cm, and good Apgar scores. She was both breastfed and bottle-fed using formula milk. There was first-degree consanguinity between the parents. No history of recurrent infections or seizures was reported. There was a family history of an elder female sibling’s death at three months of age with similar symptoms. The cause of her death was not known. On physical examination, she was found to have a doll-like face with round cheeks (Figure 1). Her vitals were as follows: heart rate of 184 beats/minute; respiratory rate of 80 cycles/minute; blood pressure of 75/52 mmHg; capillary refill of three seconds; temperature of 38.8°C; oxygen saturation of 98% on room air. She had mottled marbling. Her weight was 5 kg, height was 56 cm, and head circumference was 35 cm. Her abdomen was slightly distended and the liver was palpable 4 cm below the costal margin. The spleen was not palpable. Other systemic reviews did not reveal any abnormalities. Emergency treatment included intravenous fluids, and ceftriaxone was used. The initial blood investigations showed a C-reactive protein level of 6.9 mg/l, procalcitonin level of 8.32 ng/ml, initial peripheral white cell count of 7220/μl with a neutrophil count at 3270/μl, platelet count of 461,000/mm3, and hemoglobin level of 8.4 g/dl. Blood chemistry revealed hypoglycemia at 21 mg/dL, hypercholesterolemia, hypertriglyceridemia, hyperuricemia, hypercalcemia (Table 1), urinary calcium/creatinine ratio (UCa/UCr) at 3.28, serum 25-hydroxyvitamin D (25 OH-vit D) at 26.5 ng/ml, normal parathyroid hormone (PTH) level at 12 pg/mL, total protein at 66 g/L, serum albumin at 43.8 g/L, alkaline phosphatase at 320 U/L, serum bilirubin at 0.15 mg/dl, elevated liver enzymes (aspartate transaminase of 739 UI/l and alanine transaminase of 229 UI/l), normal coagulation profile, creatine kinase at 37 UI/L, and normal renal function. Amylase and lipase levels were not detected. Her blood gas analysis indicated severe metabolic acidosis (pH: 7.03; bicarbonate: 6 mmol/L) with high lactate (20 mmol/l).

**Table 1:** Our patient's biological findings upon admission SGOT: serum glutamic oxaloacetic transaminase; SGPT: serum glutamic pyruvic transaminase.

Laboratory parameter	Initial value	Reference range
Total cholesterol (g/L)	2.08	<1.70
Triglycerides (g/L)	12.98	<1.5
Calcium (mg/L)	137	90-110
Uric acid (mg/L)	86	25-55
Albumin (g/L)	43.8	27-41
SGOT (UI/L)	739	5-34
SGPT (UI/L)	229	5-55
Bicarbonates (mmol/L)	6	16-24
Lactate (mmol/L)	20	2-4

**Figure 2:**
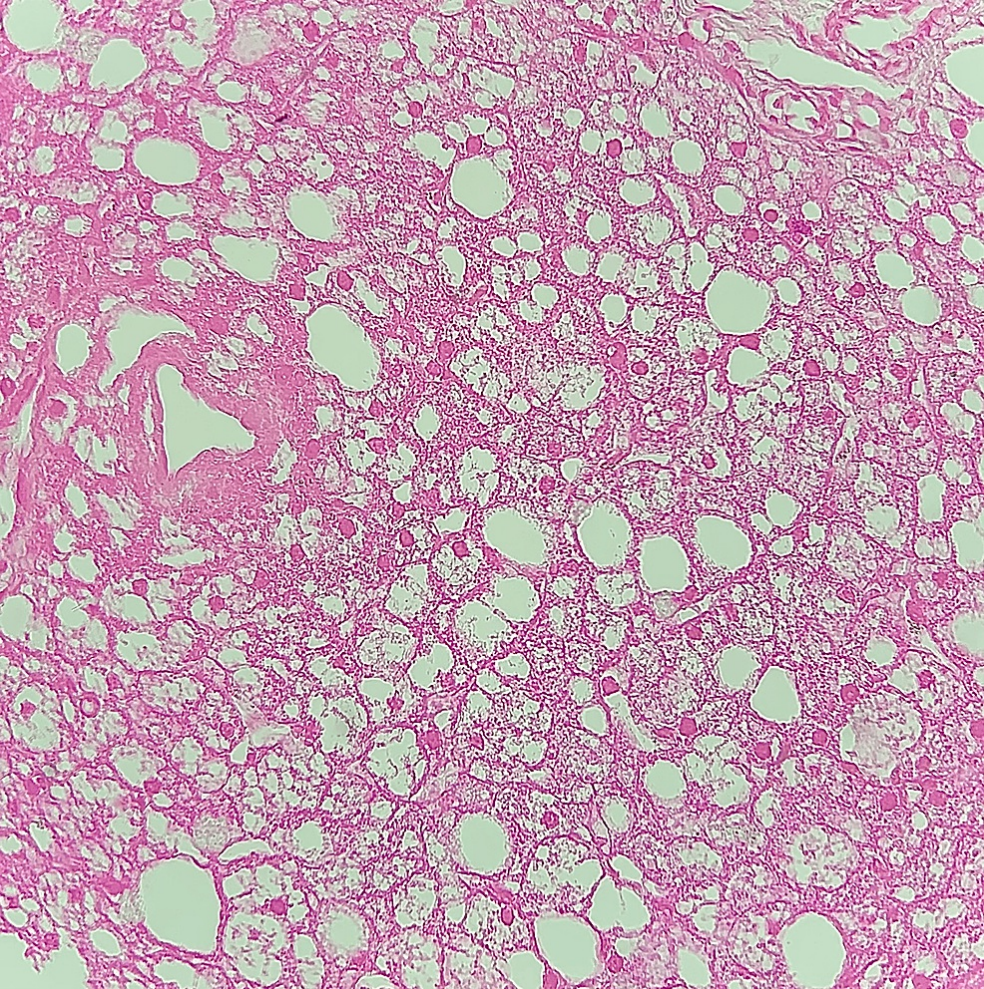
Pale and enlarged hepatocytes with prominent cytoplasmic membranes (HEx20)

**Figure 3:**
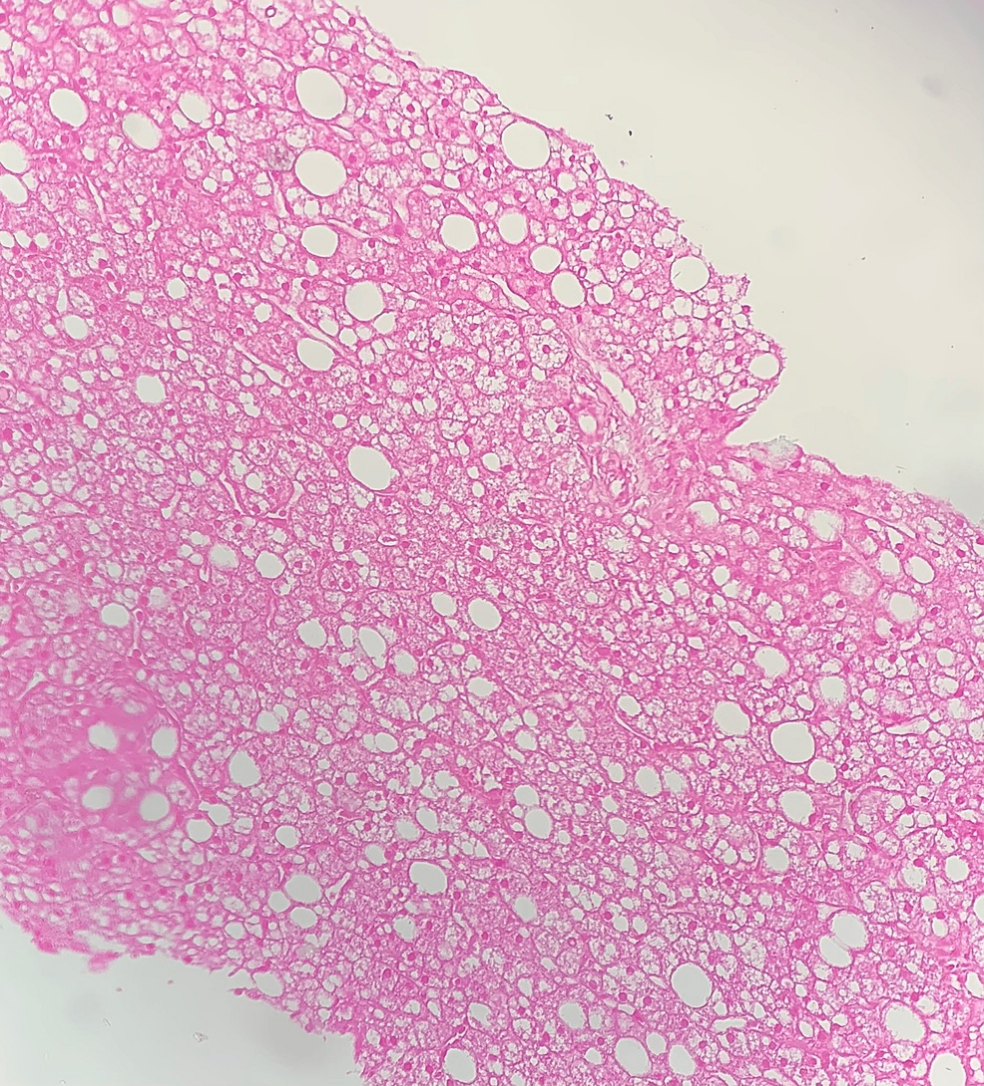
The mosaic appearance of the hepatic parenchyma associated with macrovacuolar steatosis (HEx10)

Abdominal ultrasound showed an enlarged, hyperechoic liver and grade III bilateral medullary nephrocalcinosis. Given her history and clinical presentation, as well as blood test results, a hereditary metabolic disease was suspected. A liver biopsy was performed for histopathological analysis, which was suggestive of the following features of GSD: hepatic parenchyma with 09 portal spaces with thickened trabeculae, compressing the sinusoids and presenting a mosaic-like appearance with macrovacuolar steatosis. Periodic acid-Schiff (PAS) staining was performed and returned positive, showing the presence of intra-cytoplasmic inclusions (Figures 2, 3). Enzyme study or genetic study could not be done due to unavailability. During the hospitalization, continuous glucose infusion and antibiotic treatment were given. Hypercalcemia was treated by the traditional approach with intravenous hydration and furosemide infusion. Blood glucose, blood electrolytes, lactate, and lipids were monitored. The blood gas analysis returned to normal, with gradual normalization of all other parameters. Glucose infusion was gradually stopped, and a lactose-free formula with one to two cereal tablespoons per meal was given. Our patient was discharged home after three weeks of hospitalization.

## Discussion

GSDI is an autosomal recessive disorder caused by a defect in the glucose-6-phosphatase complex, an enzyme that catalyzes the hydrolysis of glucose-6-phosphate (G6P) to glucose and inorganic phosphate (Pi). Two main subgroups of GSDI are recognized: classic type Ia GSD results from a deficiency in the activity of the glucose-6-phosphatase-a catalytic subunit (G6Pase), and type Ib GSD is caused by a defect in the glucose transporter protein-6-phosphate ( G6PC) [2]. The age of presentation varies. In the European Study on Glycogen Storage Disease Type I (ESGSD I), 50% of patients with GSD presented between one and six months, as was the case in our patient, while almost 13% presented before one month of age [4]. Occasionally, people with GSD1 present in adulthood. Carvalho et al. reported five adult patients with von Gierke’s disease, one of whom was diagnosed in adult life after developing hepatocellular adenoma [5]. GSDI patients present with hepatomegaly causing a protruding abdomen, a characteristic ‘‘doll-like’’ face with excess adipose tissue in cheeks (clinical features similar to our patients), short stature, relatively thin extremities, and chronic fatigue [3]. Symptoms could appear in the neonatal period, including hypoglycemic convulsions and lactic acidosis, and hepatomegaly may be present at birth in some cases. However, the condition is usually diagnosed when the interval between feedings increases or in the course of intercurrent illnesses responsible for symptomatic hypoglycemia [1]. Disruption in glucose homeostasis in GSD leads to secondary metabolic disturbances, most commonly hypoglycemia with concomitant lactic acidosis, increased cholesterol, triglycerides, and uric acid, and metabolic acidosis; similar findings were also observed in our patient. Increased liver enzymes may sometimes be noted early in the course of the disease, typically around the time of diagnosis. In GSD type Ib, neutrophil abnormalities are responsible for recurrent infections, which were absent in our case [3]. A rarely reported biological finding is hypercalcemia. A cross-sectional study of 23 pediatric subjects with GSDI found hypercalcemia to be detected in 78.3% of cases, an abnormality observed mostly during acute metabolic decompensation [6]. A previous report about hypercalcemia in a GSD-Ia patient who had R83H and 341delG mutations also described a three-month-old infant with hypercalcemic episodes associated with irritability and vomiting [7]. Our patient had also presented with hypercalcemia, incessant crying, and other signs of metabolic decompensation, leading us to believe that hypercalcemia should be considered as one of the problems of GSDI patients during acute attacks. It could be linked to prolonged lactic acidosis or may be pseudo hypercalcemia due to hyperlipidemia (laboratory tests sometimes reveal misleading acute hypercalcemia in hyperlipidemic serum samples) that can be seen in GSDI patients with poor metabolic control [8]. It should be emphasized that liver biopsy is not necessary when GSD is suspected, since gene sequencing is now available for individual disorders [1]. In our case, we did not dispose of such an examination, thus the diagnosis of GSDI was retained based on clinical, biochemical, and histological features. We also could not confirm the exact sub-type of GSD due to the unavailability of enzyme tests but the absence of neutropenia suggests that it is more likely a case of GSD-Ia. Management of GSDI is essentially dietary, thus, avoidance of fasting is the first line of treatment for GSDI. To prevent hypoglycemia, small frequent feedings high in complex carbohydrates are evenly distributed over 24 hours. In infancy, a soy-based, sugar-free formula or a formula that is free of sucrose, fructose, and lactose is given on demand every two to three hours. Once the baby is able to sleep longer than three to four hours at a time, and to avoid hypoglycemia during the overnight fast, options include waking the infant hours to offer feedings and monitor blood glucose or using overnight gastric feedings (OGFs) (through a nasogastric or gastrostomy tube) [1]. Young children with GSDI are at risk of a variety of nutritional deficiencies without appropriate supplements, therefore a complete multivitamin with minerals is essential [1]. There is no consensus regarding the age at which corn starch therapy should be initiated, but a trial is often introduced between six months and one year of age. General guidelines for dosing corn starch are detailed in the American College of Medical Genetics and Genomics standards and guidelines [1].

## Conclusions

Among the various metabolic abnormalities involved in GSD, this case report highlights the possibility of the presence of hypercalcemia in GSD patients, a rare biological finding that has mostly been described in other reports to be related to acute metabolic decompensation. Early detection of the disease as well as adequate dietary management improves survival and quality of life and minimizes complications.
